# Mycobiota of Acorn Worm *Saccoglossus mereschkowskii* (Hemichordata, Enteropneusta) and Its Habitats

**DOI:** 10.1134/S0012496624600568

**Published:** 2025-04-11

**Authors:** O. V. Ezhova, O. A. Grum-Grzhimaylo, Ju. A. Kokurkina, I. A. Ekimova, M. M. Gantsevich, V. V. Malakhov

**Affiliations:** 1 Department of Invertebrate Zoology, Biological Faculty, Moscow State University, 119234 Moscow, Russia; 2 Pertsov White Sea Biological Station, Biological Faculty, Moscow State University, 119234 Moscow, Russia

**Keywords:** hemichordata, marine fungi, acorn worm tissues, acorn worm burrows, bottom sediments, White Sea

## Abstract

Tissues and mucus of acorn worms are known to contain phenolic compounds, which inhibit activity of aerobic bacteria. Mycobiota of tissues, the body surface, and burrows were studied for the acorn worm *Saccoglossus mereschkowskii* (Wagner, 1885). Three fungal species were found in the plating of intestine samples. Seven fungi were isolated from samples washed out from the *S. mereschkowskii* body surface. Five species were found in burrow samples; and 15, in sediment samples from the *S. mereschkowskii* habitat. Cultures of intact specimens, gill bars, and axial organ did not reveal the growth of mycelial fungi. A potential fungistatic effect was consequently assumed for acorn worm tissues.

Enteropneusta (acorn worms) are worm-like marine animals of the phylum Hemichordata. Most acorn worms are burrowing animals and live in burrows, which are made in bottom sediments. The acorn worm body lacks any protective structures. A ciliated epithelium with abundant mucous glands coats the outer surface of the body. Mucus serves as a lubricant when acorn worms move through the sediment and strengthens the walls of their burrows [1]. Acorn worm tissues and mucus are known to contain phenolic compounds, which inhibit aerobic bacteria [2]. Mycelial fungi and yeasts inhabit marine sediments [3, 4]. Mycobiota associated with acorn worms has not been studied as of yet. In particular, it is unclear whether phenolic compounds contained in acorn worm tissues and mucus suppress marine fungi. The goal of this work was to study the marine fungi associated with acorn worms and their burrows in bottom sediments.

Our subjects were the shallow-water burrowing acorn worm *Saccoglossus mereschkowskii* (Wagner, 1885) (Harrimaniidae, Enteropneusta, Hemichordata) and fungi inhabiting the bottom sediment in *S. mereschkowskii* habitats. Acorn worms were collected in the lower littoral zone near the N.A. Pertsov White Sea Biological Station (Moscow State University) in the Kandalaksha Bay of the White Sea. Fungi were cultured on the standard wort agar medium (the wort concentration 6.5%) based on seawater and supplemented with the Ceftriaxone antibiotic to inhibit bacterial growth. Intact *S. mereschkowskii* acorn worms and worm organs that are in contact with the environment (the intestine, gill bars, and the axial organ) were placed on the culture medium. Prior to plating, the intact *S. mereschkowskii* worms were washed five times with sterile seawater. To wash the material from the *S. mereschkowskii* body surface, live acorn worms were placed individually in 2-mL Eppendof tubes with 1 mL of sterile seawater and the tubes were shaken. Washing fluid samples were plated on the culture medium. Three culture variants were used to study the mycobiota of *S. mereschkowskii* burrows: (1) a microbiological needle was passed over the walls of a burrow without impairing its integrity, and the material was plated on the culture medium; (2) a burrow was cut longitudinally in sterile seawater, and material was collected from the inner surface; and (3) a burrow was cut longitudinally in sterile marine water, and one of the halves was plated so that its inner surface was against the culture medium. Sediment samples from the *S. mereschkowskii* habitats were collected outside burrows and plated as a control. The total numbers of samples and cultures on various substrates are summarized in Table 1. Plates with cultures were incubated at 21°C. The observation period was 14 days. Fungal colonies grown on the plates were isolated in pure cultures for subsequent identification by morphological and cultural features (MCF) [5–7] or molecular genetic identification (MGI). Modern species and generic names of marine fungi were verified against the Index Fungorum database (www.indexfungorum.org).

**Table 1.** Fungi cultured from tissues, body surface material, and burrows of *S. mereschkowskii* and sediment samples from its habitats

**Specimen**	**Number** **of samples**	**Number** **of cultures per sample**	**Identified marine fungi**	Identification method*
Intact acorn worm	25	1	not found	–
Gill bar	10	1	not found	–
Axial complex	10	1	not found	–
Intestine	10	1	*Penicillium* sp.	MCF [5, 6]
			*Rhodotorula mucilaginosa* (A. Jörg.) F.C. Harrison 1927**	MGI (98.48)
			*Sarocladium kiliense* (Grütz) Summerb. 2011	MGI (99.3)
Material washed from body surface	15	4	*Emericella versicolor* (Vuill.) Pitt and A.D. Hocking, 2022	MGI (99.14)
			*Penicillium bialowiezense* K.W. Zaleski 1927	MGI (95.04)
			*Penicillium brevicompactum* Dierckx 1901	MGI (95.714)
			*Penicillium corylophilum* Dierckx 1901	MGI (98.29)
			*Penicillium roseopurpureum* Dierckx 1901	MGI (100)
			*Penicillium verrucosum* Dierckx 1901	MGI (96.62)
			Rh. mucilaginosa**	MGI (100)
Burrow	3	5	Ascomycota sp.	MGI (100)
			*Cladosporium allicinum* (Fr.) Bensch, U. Braun and Crous 2012	MGI (99.25)
			*Penicillium* sp.	MCF [5, 6]
			*Plectosphaerella plurivora* A.J.L. Phillips, Carlucci and M.L. Raimondo 2012	MGI (100)
			*Umbelopsis ramanniana* (Möller) W. Gams 2003	MGI (97.56)
Bottom sediment from habitat	1	5	*Acremonium fuci* Summerb., Zuccaro and W. Gams 2004	MGI (100)
			*Aspergillus* sp.	MCF [7]
			*Baltazaria galactina* (Fr.) Leal-Dutra, Dentinger and G.W. Griff. 2018	MGI (95.3)
			*Botrytis cinerea* Pers. 1801	MGI (96.47)
			*Cutaneotrichosporon dermatis* (Sugita, M. Takash., Nakase and Shinoda) Xin Zhan Liu, F.Y. Bai, M. Groenew. and Boekhout 2015**	MGI (97.15)
			*Penicillium* sp.	MCF [5, 6]
			*P. brevicompactum*	MGI (99.49)
			*Penicillium chrysogenum* Thom 1910	MGI (99.84)
			*Penicillium montanense* M. Chr. and Backus 1963***	MGI (92.77)
			*Penicillium rubens* Biourge 1923	MGI (99.83)
			*Penicillium thomii* Maire 1917	MCF [6]
			*Pseudeurotium bakeri* C. Booth 1961	MGI (99.43)
			Rh. mucilaginosa**	MGI (100)
			*Tolypocladium cylindrosporum* W. Gams 1971	MGI (98.81)
			*Trichoderma harzianum* Rifai 1969	MGI (98.8)

*MGI, molecular genetic identification; % of GenBank best match is shown in parentheses. MCF, identification by morphological and culture features.

**Yeast species.

***The name is no longer in use, but has no modern analog in Index Fungorum.

Genomic DNA for MGI was isolated according to a protocol of the Canadian Center for DNA Barcoding (see [8], Glass Fiber Plate DNA Extraction Protocol For Plants, Fungi, Echinoderms and Mollusks). The isolated DNA concentration was checked using NanoDrop One (Thermo Fisher, United States). Isolated DNA was used as a template to amplify the rDNA internal transcribed spacers with the primers ITS4 (5′-TCCTCCGCTTATTGATATGC-3′) and ITS5 (5′−GGAAGTAAAAGTCGTAACAAGG-3′). PCR products were detected by electrophoresis in 2% agarose gel in Tris-acetate supplemented with ethidium bromide (Amresco, United States). Ethanol precipitation with ammonium acetate was used to purify the PCR products [9]. Sanger sequencing from forward and reverse primers was carried out with a BigDye v. 3.1 kit (Thermo Fisher, United States) according to the manufacturer’s protocol, using an ABI Prism 3500 genetic analyzer (Applied Biosystems, United States) at Koltzov Institute of Developmental Biology, Russian Academy of Sciences. Chromatograms were processed and sequences from forward and reverse primers assembled using the program Geneious v. 10.1.2. Taxonomic identification was performed using the Basic Local Alignment Search Tool (www.blast.ncbi.nlm.nih.gov) и Westerdijk Fungal Biodiversity Institute database (www.wi.knaw.nl).

In total, 72 fungal isolates were obtained from the above cultures to create a working fungal collection. Identification showed that the isolates represented 26 fungal species; 20 mycelial fungi and 2 yeasts were identified to the species level (Table 1). The species *Umbelopsis ramanniana* belonged to the division Mucoromycota. *Baltazaria galactina*, *Cutaneotrichosporon dermatis,* and *Rhodotorula mucilaginosa* represented the division Basidiomycota. The other species were mostly saprotrophic and belonged to the division Ascomycota. Three isolates, including two mycelial fungi and one yeast species, were obtained from cultures of *S. mereschkowskii* intestine samples. Seven isolates, including six mycelial fungi and one yeast species, were obtained from the material washed from the *S. mereschkowskii* body surface. Six isolates of five mycelial fungal species were obtained from *S. mereschkowskii* burrow samples. A total of 46 isolates were obtained from sediment samples collected in the *S. mereschkowskii* habitats; the set included 13 mycelial fungi and 2 yeast species. The occurrences of various fungal genera in the isolates are shown in Fig. 1.

**Fig 1.**
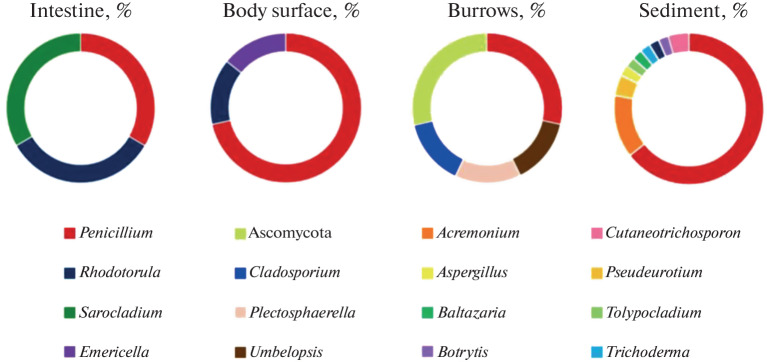
Proportions of fungal genera in isolates from the intestine, material washed from the body surface, and burrows of *S. mereschkowskii* and sediment samples collected from its habitats.

In sediment cultures (Fig. 2d), fungal colonies became detectable 5 days after plating. The mycobiota of the sediments inhabited by the acorn worm under study is comparable in diversity with that known for marine sediments of other oceanic regions [3, 4, 10–12]. Several fungi species that we found only in the sediment samples have already been detected in associations with plants and animals. For example, *Penicillium chrysogenum* had been found on algae and corals [4] and this species is widespread in soils [3], and is common on the floor of the White Sea [11]. *Cutaneotrichosporon dermatis* has been described as an inhabitant of soft corals of Brazilian reefs [13]. *Penicillium montanense* has been found on marine sponges [14]. *Pseudeurotium bakeri* has been described in association with algae [15]. *Baltazaria galactina* and *Botrytis cinerea* are widespread plant pathogens [16]. The typical marine species *Acremonium fuci* and the fungi *Penicillium thomii*, *Tolypocladium cylindrosporum,* and *Trichoderma harzianum* have been found in bottom sediments of the White Sea [11]; *P. thomii* and *T. cylindrosporum* have additionally been detected in the sediments of the Chukchi Sea [12].

In cultures from acorn worm burrows, fungi became detectable on day 7 after plating. Three fungal species were identified in the respective isolates: *Cladosporium allicinum*, *Umbelopsis ramanniana,* and *Plectosphaerella plurivora*. It is known that other species of the genus *Cladosporium* have been found on seaweed (*Cl. cladosporioides* [4]); in soils of Arkhangelsk oblast [17]; and in bottom sediments of the Barents, Cara, White, and Chukchi seas [10–12]. *U. ramanniana* is also a widespread soil species [6], and *U. isabellina* has been detected in bottom sediments of the Cara Sea [10]. *Pl. plurivora* is a nematophagous and phytopathogenic fungus; is found predominantly at equatorial, tropical, and subtropical latitudes; and inhabits decaying remains of plants, in particular, tomatoes [18, 19]. It is of interest that the mycobiota of acorn worm burrows showed a several times lower species diversity as compared with the mycobiota of the surrounding bottom sediment and was represented by other fungal species (Fig. 1, Table 1).

In cultures of the material washed from the *S. mereschkowskii* body surface (Fig. 2c), fungal colonies became detectable on day 5 after plating. Species of the genus *Penicillium* found in the cultures included *P. bialowiezense*, *P. corylophilum*, *P. roseopurpureum,* and *P. verrucosum*, which have not been mentioned in association with any animals in the available literature, and *P. brevicompactum*, which has been described as an inhabitant of anemones [4]. *Emericella versicolor* was additionally observed. *Emericella* sp. has already been detected on sponges [4]. The species *Trichoderma reesi* and *Tr. atroviride* have previously been found on sponges as well [4], while *Tr. harzianum* was detected only in sediment samples in our study. The cosmopolitan species *Tr. viride* has been found in bottom sediments of the Chukchi Sea [12]. The mycobiota of the material washed from the *S. mereschkowskii* body surface also showed low diversity and included fungi other than in the burrows and bottom sediment samples (Fig. 1, Table 1). The finding indicates that mucus produced by the *S. mereschkowskii* ectodermal epithelium to strengthen the burrow walls lack absolute fungicidal activity, but changes the environment to create the conditions that are favorable for certain species and adverse for certain others.

**Fig 2.**
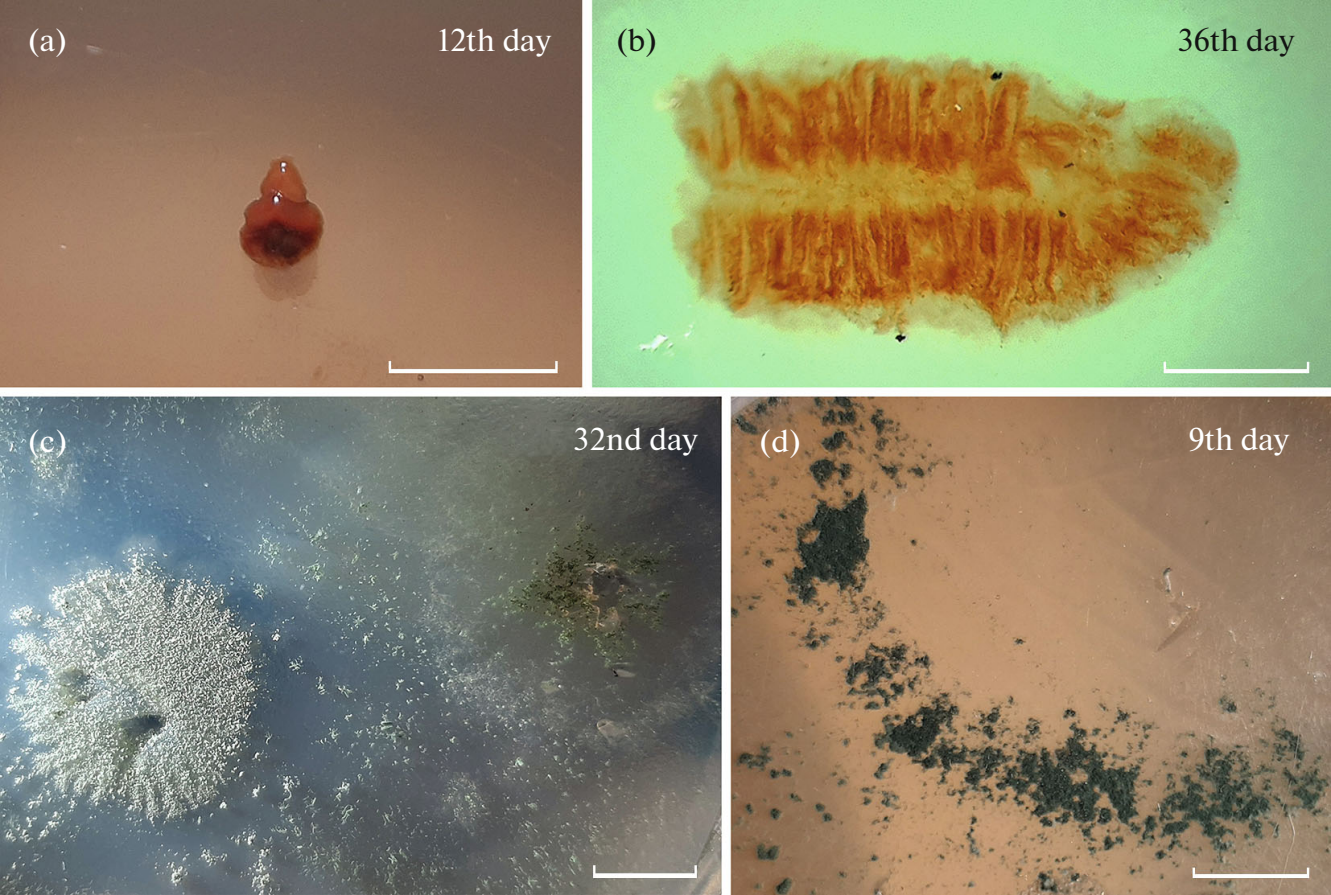
Cultures from (a) intact *S. mereschkowskii* acorn worms, (b) gill bars, (c) material washed from the *S. mereschkowskii* body surface, and (d) bottom sediment from *S. mereschkowskii* habitats. The day after plating is indicated. Bar, (a, c, d) 1 cm or (b) 1 mm.

In cultures of intestine samples, fungal colonies became detectable on day 5 after planting as well. Only three isolates were detected in the *S. mereschkowskii* intestinal contents (Table 1). Two of them were identified to the species level, and the third one was assigned to the genus *Penicillium*. Species of the genus are widespread in both marine and terrestrial ecosystems [4, 10, 11]. *Sarocladium* *kiliense* is a soil fungus and can have an endolichenic lifestyle [17, 20]; *S. strictum* is another species of the same genus and has already been detected in sediments of the Barents and White seas [10, 11]. The yeast species *Rhodotorula mucilaginosa*, which was found not only in the intestinal contents, but also in the material washed from acorn worm body surface and benthic sediment samples, is a ubiquitous soil species [3]. Thus, the fungi detected in the *S. mereschkowskii* intestine are not strictly marine fungi, and are widespread in terrestrial communities. Acorn worms are omnivorous deposit feeders, which digest organic remains as well, including fungi. This possibly explains why isolates and fungi in their intestine were far less numerous than in surrounding bottom sediments.

Mycelial fungi were not detected in cultures from intact *S. mereschkowskii* acorn worms (Fig. 2a), gill bars (Fig. 2b), and axial complexes within the observation period. Lack of fungal growth and reproduction possibly suggests fungistatic activity for acorn worm tissues.
